# Traditional and Innovative Processing of Georgian Myrobalan Plum (*P. cerasifera Ehrh*): Effects on Phytochemical Content

**DOI:** 10.1002/fsn3.71458

**Published:** 2026-01-26

**Authors:** Jeiran Putkaradze, Maia Vanidze, Sopio Ghoghoberidze, Ruslan Davitadze, Aleko Kalandia

**Affiliations:** ^1^ Department of Chemistry, Faculty of Natural Sciences and Health Care Batumi Shota Rustaveli State University (BSU) Batumi Georgia; ^2^ Qlab Private Company, Research and Development Quality Control and Testing Services Thessaloniki Greece

**Keywords:** anthocyanins, antioxidant activity, mass spectrometry, phenolic compounds, *Prunus*

## Abstract

This study provides a comprehensive chemical and functional characterization of Georgian Myrobalan plum ( *Prunus cerasifera* Ehrh.), a traditional fruit known locally as “tkemali.” Using advanced analytical methods including UPLC–PDA–MS and LC–ESI–MS, 34 phenolic compounds were identified, encompassing anthocyanins, hydroxycinnamic acids, flavan‐3‐ols, and flavonol glycosides. Cyanidin‐based pigments, particularly cyanidin‐3‐O‐rutinoside and cyanidin‐3‐O‐galactoside, dominated the anthocyanin profile, while resveratrol was tentatively detected for the first time in this species. Organic acid analysis revealed malic acid as the principal acidulant, contributing to the fruit's characteristic tartness and strong buffering capacity. Comparative processing studies demonstrated that traditional high‐heat methods (jam, open‐pan concentrate) caused severe degradation of heat‐labile phenolics and anthocyanins, with losses exceeding 90%, and significantly reduced antioxidant capacity. In contrast, innovative low‐temperature technologies such as freeze‐drying and rotary vacuum concentration preserved up to ninefold higher anthocyanin and sevenfold higher phenolic acid levels, enhancing the nutritional and functional quality of processed products. The fruit also exhibited high potassium content, reinforcing its nutritional value. These findings highlight *P. cerasifera* as a rich source of bioactive compounds and underscore the potential of non‐thermal processing methods to retain its phytochemical integrity. The study establishes a scientific foundation for the valorization of Georgian tkemali as both a cultural product and a modern functional food ingredient.

## Graphical abstract

This study provides the first comprehensive phytochemical characterization of Georgian *Prunus cerasifera* (tkemali) fruits and compares the effects of traditional high‐heat processing with innovative low‐temperature methods on bioactive compound retention. We identified 33 phenolic compounds and found that freeze‐drying and vacuum concentration preserved up to ninefold higher anthocyanin and sevenfold higher phenolic acid levels compared with jam and open‐pan boiling. These findings demonstrate that modern low‐temperature technologies significantly improve the nutritional and functional quality of tkemali‐based products.

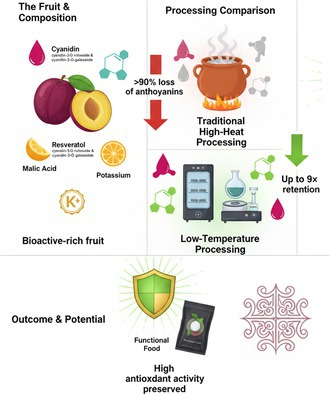

**Abbreviations**
AA_50_
: sample mass (mg) required to inactivate 0.1 mM DPPH by 50%
ANOVA: analysis of variance
AU: absorbance units
Ca^2+^
: calcium ion
Cy‐gal: cyanidin‐3‐O‐galactoside
Cy‐rut: cyanidin‐3‐O‐rutinoside
DPPH: 2,2‐diphenyl‐1‐picrylhydrazyl radical
DW: dry weight
ESI: electrospray ionization
FW: fresh weight
HPLC: high‐performance liquid chromatography
IC_50_
: inhibitory concentration required for 50% radical reduction
IC: ion chromatography
K^+^
: potassium ion
LC–MS: liquid chromatography–mass spectrometry
Mg^2+^
: magnesium ion
PDA: photodiode array detector
RT: retention time (min)
SD: standard deviation
SEM: standard error of mean
TAC: total anthocyanin content
TFC: total flavonoid content
TPC: total phenolic content
°Brix: total soluble solids (% w/v)

## 1 Introduction

Interest in raw materials from the *Prunus* family rich in biologically active compounds, as well as products obtained from it, is quite high all over the world. Biologically active compounds of fruits, bones and leaves have been studied (Fratianni et al. 2021; Hu et al. 2021; Jawad et al. 2022; Liaudanskas et al. 2020; Saraswathi et al. 2020; Sottile et al. 2023; Valderrama‐Soto et al. 2021; Wang et al. 2012). A variety of *Prunus cerasifera* Ehrh. has been of the greatest research interest recently. Many biologically active compounds have been identified in its fruits (Celik et al. 2017; Moscatello et al. 2019), and the chemical composition of new selective forms of myrobalan ( *P. cerasifera* L.) and their effect on the taste characteristics of fruits have been studied (Saridaş et al. 2016; Sottile et al. 2023). In several varieties, there has been examined the content of carbohydrates, organic acids and other compounds during fruit ripening (Moscatello et al. 2019; Saridaş et al. 2016). The quantitative content of phenolic compounds, organic acids (Gündüz and Saraçoğlu 2012; Moscatello et al. 2019), the chemical composition of hybrid varieties (Uğur et al. 2021), the content of fat and fatty acids of *P. cerasifera* skin (Saeed et al. 2021), as well as the possibility of obtaining biodiesel (Górnaś et al. 2016; Saeed et al. 2021), and the antioxidant and antibacterial activity of fruits (Gündüz and Saraçoğlu 2012; Saraswathi et al. 2020). Using the HPC‐DAD/ESI‐MS method, the content of anthocyanins in the leaves of *P. cerasifera* was studied; several phenolic acids were identified (Chen et al. 2018). The resulting preparations were used to obtain a nanopreparation (Jaffri and Ahmad 2018). A study of *P. cerasifera* tree gum showed that the dominant substances are arabinose and galactose (Shi et al. 2019).

*P. cerasifera* is a widespread plant in Georgia. There can be found both wild and cultivated species of this plant (Putkaradze, Vanidze and Kalandia 2025). In different corners of Georgia, people prepare various products from the fruits of *P. cerasifera* according to different recipes: sauce, tklapi, korao, jam and others. Nevertheless, in the numerous literature sources reviewed by us, only works published in 1981 deal with studies of *P. cerasifera* fruits growing in Georgia, as well as their morphological diversity (Kharadze et al. 2018).

Despite the popularity of *P. cerasifera* and its fruit‐derived products, there has been no study on the bioactive compounds of *Prunus* fruits and the chemical composition of its products, nor the changes that occur during processing in Georgia. The plant of *P. cerasifera* can grow as a tree‐like shrub with a highly branched structure and may have prickly or thornless characteristics. Its fruits can be round, oblong‐round, or ovoid in shape. It is known to grow well in both cultivated and uncultivated areas, and it tends to form a dense, spiny mass along roads, canals, and wetlands on the leeward side.

The present study aims to investigate the chemical composition of wild‐growing and cultivated ecotypes of *P. cerasifera* in Georgia and to characterize the effects of traditional fruit processing methods on the stability and profile of key bioactive compounds.

## 2 Materials and Methods

### 2.1 Chemicals and Reagents

All chemicals, solvents, and reagents utilized throughout this study were of analytical or HPLC grade to ensure the highest accuracy and reproducibility of experimental results. The mobile phases for chromatographic separations were prepared using HPLC‐grade methanol (Roth, Germany, Art.‐Nr. 8388.6), acetonitrile (CAS No. 75‐05‐8, Germany), and formic acid (CAS No. 64‐18‐6, Finland). Key reagents for the various spectrophotometric analyses included Folin–Ciocalteu's phenol reagent (Sigma‐Aldrich, Cat. No. 102664886) for total phenolic content determination, the stable radical 2,2‐diphenyl‐1‐picrylhydrazyl (DPPH) (Sigma‐Aldrich) for assessing antioxidant capacity, and vanillin (Sigma‐Aldrich, Cat. No. V1104‐100G). For the precise identification and quantification of target analytes, certified analytical standards were procured from reputable suppliers. These included chlorogenic acid (Sigma‐Aldrich, Lot No. SLBJ3632V), caffeic acid (Roth, Germany, Art.‐Nr. 5869.3), and (+)‐catechin hydrate (Biosynth, FC30661). All other necessary chemicals, such as anhydrous sodium carbonate, sodium nitrite, aluminum chloride, and sodium hydroxide, were also of analytical grade. Ultrapure water (18.2 MΩ·cm), generated by a laboratory water purification system, was used exclusively for preparing all aqueous solutions, buffers, and mobile phase dilutions.

### 2.2 Plant Material

**FIGURE 1:**
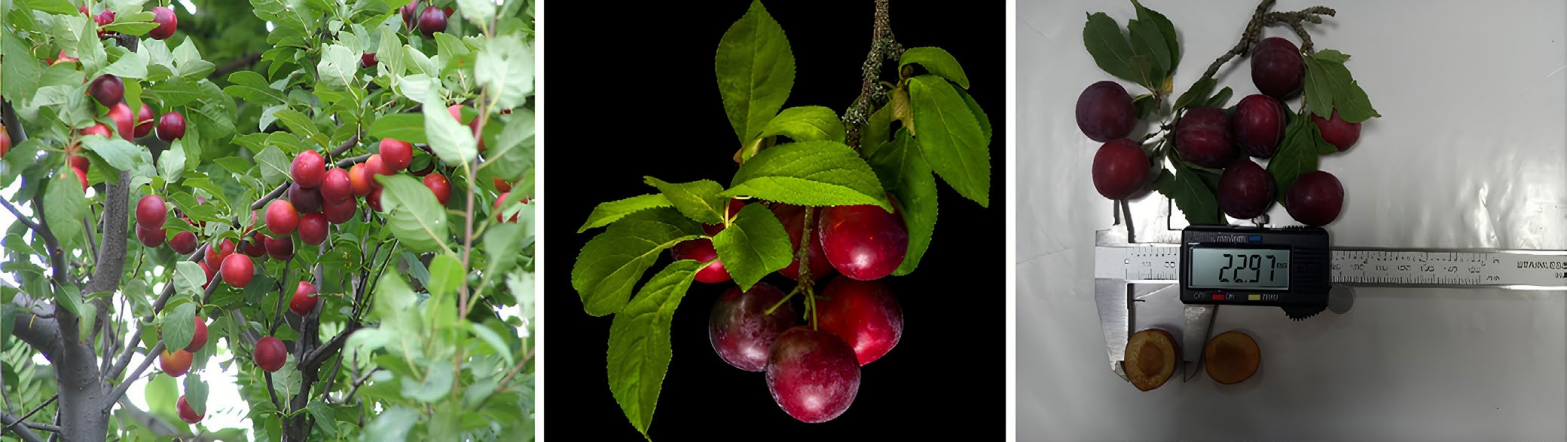
*P. cerasifera Ehrh*.

The plant material for this investigation consisted of wild‐growing Myrobalan plum ( *P. cerasifera* ), locally known as Tkemali, a member of the Rosaceae family. Fruits were collected over the harvesting seasons from 2016 to 2024 from a specific geographical location in the Adjara region of Western Georgia, specifically in the Khulo municipality (41°38′47″ N, 42°18′40″ E). To ensure consistency and relevance for consumption, all fruits were carefully harvested at the stage of optimal consumer maturity, judged by their characteristic color, firmness, and flavor profile (Figure 1). The botanical identity of the plant material was confirmed prior to analysis.

### 2.3 Samples Preparation

Upon collection, the 20 kg batch of fresh plant material was immediately transported to the laboratory. The fruits were meticulously sorted by hand to remove any extraneous materials, such as leaves and stalks (approximately 1.5%), ensuring only sound, ripe plums were retained for analysis. To preserve phytochemical integrity and inhibit enzymatic degradation, the cleaned samples were promptly frozen and stored at −20°C until further processing.

### 2.4 Processing Methods

#### 2.4.1 Juice Extraction: Cold Pressing vs. Hot Extraction

Primary juice was extracted from the composite sample using two fundamentally different protocols to assess the impact of thermal treatment at the initial stage.

The cold pressing method was implemented as a non‐thermal protocol aimed at preserving the native state of heat‐sensitive compounds. For this procedure, thawed plums from the composite batch were first mechanically pitted to remove the stones. The resulting pulp and skin mass was then immediately processed using an Angel Juicer 7500. This twin‐gear triturating juicer was specifically chosen for its low‐speed operation (82 RPM), which minimizes both the incorporation of atmospheric oxygen and the generation of frictional heat, two key factors known to initiate the degradation of phenolic compounds and vitamins. The raw juice extracted was passed through a fine‐mesh sieve and subsequently filtered through four layers of cheesecloth to remove larger particulate matter, yielding a clarified juice ready for analysis.

The hot extraction method simulated a common industrial practice involving thermal pretreatment. Whole, unpitted plums from the composite batch were blanched by complete immersion in a thermostatically controlled water bath set to 90°C–95°C for a precisely timed duration of 4 min. The primary goals of this step were to inactivate endogenous enzymes like polyphenol oxidase and to thermally soften the fruit's cellular structure, thereby facilitating a more efficient release of juice and pigments. Immediately after the 4‐min blanching period, the fruits were removed and plunged into an ice‐water bath to rapidly quench the thermal process, preventing over‐cooking and degradation. Once cooled, the blanched plums were mechanically pressed using a hydraulic press to separate the juice from the pomace.

#### 2.4.2 Preparation of Plum Products

The juices and pomace derived from the composite sample were further processed into a range of traditional Georgian food products, with each product being made via both a traditional, high‐heat method and a modern, low‐temperature alternative.

##### 2.4.2.1 *Korao* (Concentrate)

The juice was concentrated to approximately 60 °Brix using two methods. The traditional approach involved open pan boiling in a stainless‐steel vessel over a direct heat source, with continuous stirring until the target concentration was reached. The modern method employed a rotary vacuum evaporator operating at a low temperature of 40°C–45°C and a reduced pressure of < 100 mbar, a technique designed to preserve volatile compounds and prevent thermal degradation.

##### 2.4.2.2 *Tklapi* (Fruit Leather)

The residual fruit pulp was used to prepare fruit leather. For traditional *tklapi*, the pulp was homogenized into a fine slurry, spread into thin (3–4 mm) layers on wooden boards, and left to dry outdoors under direct sunlight for 3–5 days, depending on weather conditions. For the innovative method, the slurry was spread onto stainless steel trays and dried in a cabinet freeze‐dryer, where water was removed by sublimation under a high vacuum.

##### 2.4.2.3 Jam

A traditional high‐sugar jam was prepared by combining fruit pomace with sucrose in a 1:1 ratio by weight. The mixture was cooked in an open stainless‐steel pot over medium heat with constant stirring until it reached a final soluble solids content of 65 °Brix, as measured by a digital refractometer.

##### 2.4.2.4 *Satsebeli* (Sauce)

The plum sauce was created by cooking the juice and pulp mixture. A standardized blend of traditional Georgian spices (coriander, garlic, savory, and red pepper) was added, and the mixture was simmered until it reached a pre‐determined viscosity. Two distinct formulations were prepared for analysis: “Sauce A” represents standard preparation, while “Sauce C” was prepared with a higher concentration of spices and cooked for a longer duration to achieve a thicker consistency.

### 2.5 Analytical Methods

#### 2.5.1 Isolation and Identification and of Phenolic Compounds

The total anthocyanin content (TAC) of *P. cerasifera* samples was determined spectrophotometrically according to the pH differential method described by (Giusti and Wrolstad 2001) with slight modifications. Fresh or processed samples (1 g) were homogenized and extracted with acidified methanol (methanol: water: formic acid, 70:29:1, v/v/v) under dark conditions at 4°C for 24 h. The extracts were centrifuged at 10,000 rpm for 10 min, and the supernatant was collected for absorbance measurement.

The total anthocyanin content (TAC) of the samples was determined spectrophotometrically. Absorbance was measured at 520 nm against a deionized water blank using a UV–Vis spectrophotometer. TAC was calculated using the following equation:

$$\mathrm{TAC}\;\mathrm{mg}/g\;\left(\mathrm{FW}\right)=\left(A_{520}\right)\times \left(M_{w}\times \mathrm{Df}\times V\right)/\left(\varepsilon \times L\times m\right)$$

where A_520_ is the absorbance reading at 520 nm, Mw is the molecular weight of cyanidin‐3‐O‐glucoside (C3G; 449.2 g/mol), *Df* is the dilution factor, ε is the molar extinction coefficient of C3G (26,900 L mol^−1^ cm^−1^), *L* is the cuvette path length (1 cm), m is the sample mass (g), and V is the solvent volume (mL).

TAC can also be expressed on a dry weight basis using the equation:

$$\mathrm{TAC}\;\mathrm{mg}/g\;\left(\mathrm{DW}\right)=\mathrm{TAC}\;\mathrm{mg}/g\left(\mathrm{FW}\right)/W\times 100$$

where W represents the moisture content (%) of the sample.

Identification of phenolic compounds—including anthocyanins, flavonoid glycosides, and phenol carbon acids—was performed using Ultra Performance Liquid Chromatography with photodiode array and mass spectrometry detection (UPLC–PDA–MS) on a Waters Acquity H‐class system (Quaternary Solvent Manager, Sample Manager–FTN, PDA Detector, and QDa Mass Detector). The chromatographic separation for non‐anthocyanin phenolics was carried out using a BEH C18 column (1.7 μm) with Solvent 1 (0.2% formic acid in water) and Solvent 2 (acetonitrile) under gradient elution (starting at 5% solvent 2), a flow rate of 0.3 mL/min, and a column temperature of 40°C. The MS was operated in negative ESI mode with a scan range of 100–1200 Da, spray voltage of 0.8 kV, capillary voltage of 1.5 kV, cone voltage (CV) of 5–40, and a probe temperature of 500°C. Anthocyanins were analyzed under the same conditions but using MeOH: deionized water with 2% formic acid as the eluent and positive ESI mode. The PDA UV–Vis spectra were recorded across 215–500 nm (Putkaradze et al. 2023; Smailagić et al. 2019).

Compound identification was based on the evaluation of characteristic fragmentation patterns and adduct ion formation observed in the mass spectra, together with the UV–Vis absorption maxima specific to each phenolic class. Structural assignments were verified using authentic reference standards (malvidin‐3‐O‐glucoside, rutin, chlorogenic acid; Sigma‐Aldrich), the METLIN spectral database, and published reference data (Smith et al. 2005).

#### 2.5.2 Quantification of Organic Acids and Carbohydrates

The quantification of major organic acids and carbohydrates was performed using two distinct high‐performance liquid chromatography (HPLC) systems. For organic acid analysis, samples were prepared by adding 96% ethanol (1:1, v/v) to precipitate pectins, followed by centrifugation. The supernatant was then diluted with the mobile phase. Solid product analysis required a prior extraction in 0.1% aqueous phosphoric acid (1:10, w/v). Chromatographic separation of L‐ascorbic acid, citric acid, and malic acid was carried out on a Shodex KC‐811 column (300 × 8 mm) under isocratic elution with 0.1% phosphoric acid as the mobile phase. Detection was performed with a UV–Vis 2489 detector set at 254 nm for ascorbic acid and 214 nm for citric acid, while malic acid was quantified via UPLC‐MS. For carbohydrate analysis, a Waters HPLC system equipped with a 1525 Binary Pump and a Refractive Index (RI) detector was used. Sugars were separated on a carbohydrate column (Merck; 250 × 4.6 mm) maintained at 40°C, with an isocratic mobile phase of 80% acetonitrile in water (v/v). In all cases, samples were filtered through a 0.45 μm syringe filter prior to injection. Quantification was based on external calibration curves constructed from certified standards, with results adjusted for dilution factors (Doyon et al. 1991).

#### 2.5.3 Determination of Cations

The concentration of major cations in the plum juice was quantified using a dedicated ion chromatography (IC) system. The instrumental setup consisted of a Waters 1515 isocratic HPLC pump coupled with a Waters 432 Conductivity Detector. Sample preparation began by precipitating pectic substances with an equal volume of 96% ethanol, followed by centrifugation at 10,000 rpm for 10 min to remove the insoluble fraction. The clear supernatant was then diluted tenfold with deionized water (total dilution factor = 20). Before injection, all samples were filtered through 0.45 μm PTFE syringe filters to eliminate residual particulates. Chromatographic separation of K^+^, Mg^2+^, and Ca^2+^ ions was carried out on an IC‐Pak Cation M/D column (4.6 × 150 mm) maintained at 35°C under isocratic elution with 3 mM HNO₃ containing 0.1 mM EDTA as the mobile phase, delivered at a flow rate of 1.0 mL min^−1^.

The conductivity detector was operated in negative polarity mode, with a base sensitivity of 2000 μS cm^−1^ and an integrator sensitivity of 0.01 μS cm^−1^. Quantification was based on external calibration curves constructed from certified single‐element standards, with all concentrations corrected for dilution factors (Trifirò et al. 1996).

#### 2.5.4 Determination of Antioxidant Activity

Antioxidant activity was assessed using the DPPH (2,2‐diphenyl‐1‐picrylhydrazyl) radical scavenging assay. A 1.0 mL aliquot of each extract was mixed with 3.0 mL of 0.1 mM DPPH methanolic solution and incubated in the dark at room temperature for 15 min. Absorbance was then measured at 517 nm using a UV–Vis spectrophotometer. The radical scavenging capacity of the sample was expressed as the percentage of DPPH inhibition (In%), calculated using the following equation:

$$\text{In}\%=\frac{\mathrm{Ac}-\mathrm{As}}{\mathrm{Ac}}\times 100$$

where In%, represents the inhibition of 0.1 mm DPPH; A_C_, is the absorbance of the 0.1 mm DPPH ethanolic solution; and A_S_, is the absorbance of the reaction mixture containing the 0.1 mm DPPH ethanolic solution and the sample extract.

The extract concentration required to achieve 50% inhibition of the 0.1 mM DPPH radical (IC_50_) was calculated using the following equation:

$${\mathrm{IC}}_{50}=\frac{m\times V\times F}{\text{In}\%50}\times 100$$

where *m* is the mass of the tested sample (mg), *V* is the volume of the sample extract (mL), *F* is the dilution factor, and In%_50_ represents the percentage of DPPH inhibition within the 45%–55% range used to interpolate the IC_50_ value (Gulcin and Alwasel 2023; Sottile et al. 2023).

#### 2.5.5 Determination of Total Flavonoid Content

A 1.0 mL aliquot of the extract was diluted to 100 mL with 50% (v/v) ethanol. From this solution, 1.0 mL was transferred into a 10 mL volumetric flask, to which 5.0 mL of distilled water and 0.3 mL of 5% (w/v) NaNO_2_ were added. After 5 min, 0.3 mL of 10% (w/v) AlCl_3_ was introduced, followed by an additional 6 min incubation. Subsequently, 2.0 mL of 1 N NaOH was added, and the volume was adjusted to the mark with distilled water. The absorbance was measured at 510 nm using a UV–Vis spectrophotometer (Datuashvili et al. 2025; Jurić et al. 2025).

#### 2.5.6 Determination of Total Phenolic Content

Five grams of the test sample were cold extracted with 50% (v/v) ethanol, and the final extract volume was adjusted to 100 mL. From this solution, 1.0 mL was transferred into a 10 mL volumetric flask, followed by the addition of 5.0 mL of distilled water, 1.0 mL of Folin–Ciocalteu reagent, and 10.0 mL of sodium carbonate solution (7.5% w/v). The mixture was then brought to volume with distilled water, thoroughly mixed, and allowed to stand for 1 h at room temperature for color development. Absorbance was measured at 750 nm using a 1 cm path length cuvette against a reagent blank prepared with the corresponding extractant (Alp 2017; Datuashvili et al. 2025; Raposo et al. 2024).

#### 2.5.7 Determination of Titratable Acidity, pH, and Soluble Solids Content

Titratable acidity was determined by titrating the sample with 0.1 M NaOH to an endpoint of pH 8.3, using phenolphthalein as an indicator. Results were expressed as malic acid equivalents (g malic acid per 100 g sample). The pH of each sample was measured directly using a calibrated digital pH meter. The soluble solids content (°Brix) was measured with a digital refractometer (Barać et al. 2022; Datuashvili et al. 2025).

#### 2.5.8 Quantitative Determination of Leucoanthocyanins (TLC Method)

A 1.0 mL aliquot of the extract was mixed with 8.0 mL of leucoanthocyanidin reagent (n‐Butanol‐HCl, 475:25 mL). For each sample, two parallel aliquots were prepared: one unheated (control) and one heated at 100°C for 40 min. After incubation, absorbance was measured at 550 nm using a UV–Vis spectrophotometer. The unheated aliquot served as the control for the heated sample. Results were expressed as mg g^−1^ dry weight (DW) using a calibration curve constructed with cyanidin‐3‐O‐glucoside standard (Chen et al. 2018; Datuashvili et al. 2025; Mannino et al. 2021).

### 2.6 Statistical Analysis

Standard error was calculated for each data point using Microsoft Excel. Confidence interval *p* ≤ 0.05 (Surmanidze et al. 2024).

## 3 Results

### 3.1 Morphology and Baseline Composition of Raw Fruit

#### 3.1.1 Fruit Morphology and Size

The fruits of *P. cerasifera*, locally known as wild *tkemali*, demonstrated substantial morphometric and phenotypic variation across the four ecotypes sampled from the Khulo district in Adjara, Georgia (Table 1). The observed diversity encompassed both shape and pigmentation: fruits were generally elongated or oblong‐rounded, occasionally nearly spherical, with pericarp colors ranging from bright red to dark burgundy. All ecotypes shared the typical sweet–sour sensory profile, reflecting the high organic acid content balanced by moderate sugar accumulation.

**TABLE 1:** *P. cerasifera* Ehrh. fruit characterization.

Fruit	Shape	Color	Taste	Size (mm)		Mass (g)	Volume (mL)
				Length	Width		
Wild red tkemali (Khulo Danisparauli 07.08)	Elongated	Dark red	Sweet–sour	23.8 ± 0.9	20.6 ± 0.8	6.3 ± 0.2	6.1 ± 0.2
Wild red tkemali (Khulo 24.09)	Oblong‐rounded	Red	Sweet–sour	26.4 ± 1.1	27.4 ± 1.1	10.2 ± 0.35	10.0 ± 0.4
Wild dark red tkemali (Khulo 24.09)	Elongated	Dark red	Sweet–sour	26.1 ± 0.8	20.8 ± 0.6	6.5 ± 0.23	7.0 ± 0.3
Wild dark red (Khulo Gorjomi, mountain Mkvirala)	Round	Dark red	Sweet–sour	21.28 ± 0.7	20.48 ± 0.7	5.3 ± 0.20	5.2 ± 0.2

*Note:* Values represent mean ± SD (*n* = 3).

Average fruit length ranged from 21.28 ± 0.7 mm (Mkvirala) to 26.4 ± 1.1 mm (Khulo 24.09 red), while width ranged between 20.48 ± 0.7 mm and 27.4 ± 1.1 mm. The mean fruit mass varied from 5.3 ± 0.20 g to 10.2 ± 0.35 g, corresponding to volumes of 5.2 ± 0.2 mL to 10.0 ± 0.4 mL. These metrics classify *P. cerasifera* within the small‐fruited plum group, consistent with its wild genetic background.

Morphological variation correlated with altitude and microclimate: the Khulo 24.09 ecotype, originating from a mid‐altitude, moderately humid zone, produced the largest and juiciest fruits, whereas the Mkvirala mountain ecotype, collected at higher elevation, exhibited smaller, denser fruits with more intense pigmentation. Such differentiation likely reflects ecophysiological adaptation to temperature gradients, influencing fruit cell wall elasticity and metabolite concentration. These findings corroborate prior reports that mountainous *P. cerasifera* populations develop compact fruits with elevated biochemical density as an adaptive response to cooler ripening conditions [24].

Fruits were harvested from three individual trees (~100 kg total), and a 10 kg composite sample was prepared for analysis.

#### 3.1.2 Organic Acids in Fresh Juice

The organic acid profile of *P. cerasifera* juice, determined by UPLC–MS, revealed a composition dominated by malic acid, accompanied by smaller amounts of quinic and citric acids (Table 2).

**TABLE 2:** The content of organic acid in the fruit and juice in different varieties of *P. cerasifera* Ehrh.

Sample name	Quinic acid %	Citric acid %	Malic acid %	Total acid %
Wild red tkemali (Khulo Danisparauli 07.08)	0.64 ± 0.020	0.03 ± 0.0009	2.21 ± 0.070	3.2 ± 0.102
Wild red tkemali (Khulo 24.09)	0.48 ± 0.016	0.06 ± 0.0002	2.52 ± 0.085	3.4 ± 0.115
Wild dark red tkemali (Khulo 24.09)	0.42 ± 0.015	0.09 ± 0.003	2.55 ± 0.096	3.4 ± 0.129
Wild dark red (Khulo Gorjomi, mountain Mkvirala)	0.53 ± 0.021	0.03 ± 0.001	2.32 ± 0.092	3.2 ± 0.112

*Note:* Values are mean ± SD (*n* = 3), expressed on juice basis (% w/v). Total acids represent the sum of individual quantified organic acids.

Quantitatively, malic acid ranged from 2.21% ± 0.070% to 2.55% ± 0.096% (w/v), representing roughly 69%–75% of total acidity, confirming its role as the principal acidulant. Quinic acid contributed 0.42%–0.015%, while citric acid was present only in trace quantities (0.03%–0.001%). The total titratable acidity of the juice reached 3.2%–3.4%, a value comparable to or slightly exceeding that of cultivated *Prunus domestica* and *P. salicina* , underscoring the naturally acidic character of wild tkemali.

**FIGURE 2:**
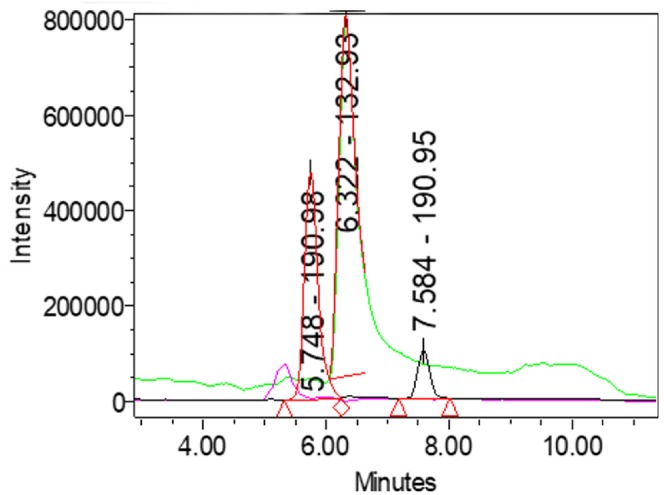
UPLC–MS chromatogram of organic acids in *P. cerasifera* juice.

The chromatographic trace (Figure 2) displayed three well‐resolved peaks: quinic acid (RT≈5.7 min), citric acid (RT≈6.3 min), and malic acid (RT≈7.5 min), with malic acid exhibiting the highest response intensity. UV detection confirmed characteristic absorbance maxima near 210–254 nm, consistent with the spectra of authentic standards.

The high malic/quinic ratio differentiates Georgian *P. cerasifera* from Central European plum cultivars, where citric acid often predominates. This trait not only contributes to the sharp, refreshing flavor typical of *tkemali* sauces but also enhances product stability, since malic acid confers stronger microbial inhibitory and buffering properties than citric acid (Ji et al. 2023).

From a physiological standpoint, the elevated malic acid content likely arises from the dominance of the malate–fumarate pathway under the cool‐temperate climatic regime, promoting accumulation of malate as a carbon sink during fruit maturation (Zhan et al. 2023).

#### 3.1.3 | Carbohydrates and Soluble Solids

The carbohydrate composition of the fresh juice (Table 3) comprised primarily glucose, fructose, and sucrose, with glucose being the predominant monosaccharide across all ecotypes. Glucose concentrations ranged from 5.50 ± 0.21 g L^−1^ to 7.18 ± 0.27 g L^−1^, fructose from 1.53 ± 0.05 g L^−1^ to 2.49 ± 0.08 g L^−1^, and sucrose from 0.25 ± 0.01 g L^−1^ to 1.73 ± 0.07 g L^−1^. The total soluble sugar content therefore varied between 8.72 ± 0.31 g L^−1^ and 10.00 ± 0.32 g L^−1^, corresponding to 9.6 ± 0.3–10.7 ± 0.4°Brix.

**TABLE 3:** Carbohydrate composition of wild *P. cerasifera* (tkemali) juices.

Sample	Fructose (g L^−1^)	Glucose (g L^−1^)	Sucrose (g L^−1^)	Total sugars (g L^−1^)	°Brix
Wild red tkemali (Khulo Danisparauli, 07.08)	1.70 ± 0.05	6.77 ± 0.24	0.25 ± 0.01	8.72 ± 0.31	9.6 ± 0.3
Wild red tkemali (Khulo, 24.09)	1.53 ± 0.05	7.18 ± 0.27	1.28 ± 0.04	10.00 ± 0.32	10.5 ± 0.3
Wild dark‐red tkemali (Khulo, 24.09)	1.90 ± 0.07	6.29 ± 0.20	0.77 ± 0.02	8.97 ± 0.36	9.9 ± 0.3
Wild dark‐red (Khulo Gorjomi, mountain Mkvirala)	2.49 ± 0.08	5.50 ± 0.21	1.73 ± 0.07	9.72 ± 0.33	10.7 ± 0.4

*Note:* Values are mean ± SD (*n* = 3).

The glucose > fructose ≫ sucrose hierarchy typifies non‐climacteric, acid‐dominant stone fruits and indicates efficient sucrose hydrolysis during ripening. The moderate °Brix values confirm the balanced sweetness of wild plums, while the sugar‐to‐acid ratio (≈2.8–3.1) explains the characteristic sweet–sour organoleptic equilibrium valued in traditional Georgian sauces and preserves. The Mkvirala mountain ecotype exhibited the highest °Brix (10.7 ± 0.4), suggesting delayed sugar metabolism and concentration effects during slow ripening under cooler temperatures.

From a technological perspective, such compositional equilibrium is advantageous for juice and concentrate production, reducing the need for artificial acidification or sweetening. It also provides a biochemical fingerprint for authenticity verification of *tkemali* products based on the glucose/malic acid ratio, which could serve as a marker of geographic and genetic origin.

### 3.2 Anthocyanin Composition and Chromatographic Identification

**FIGURE 3:**
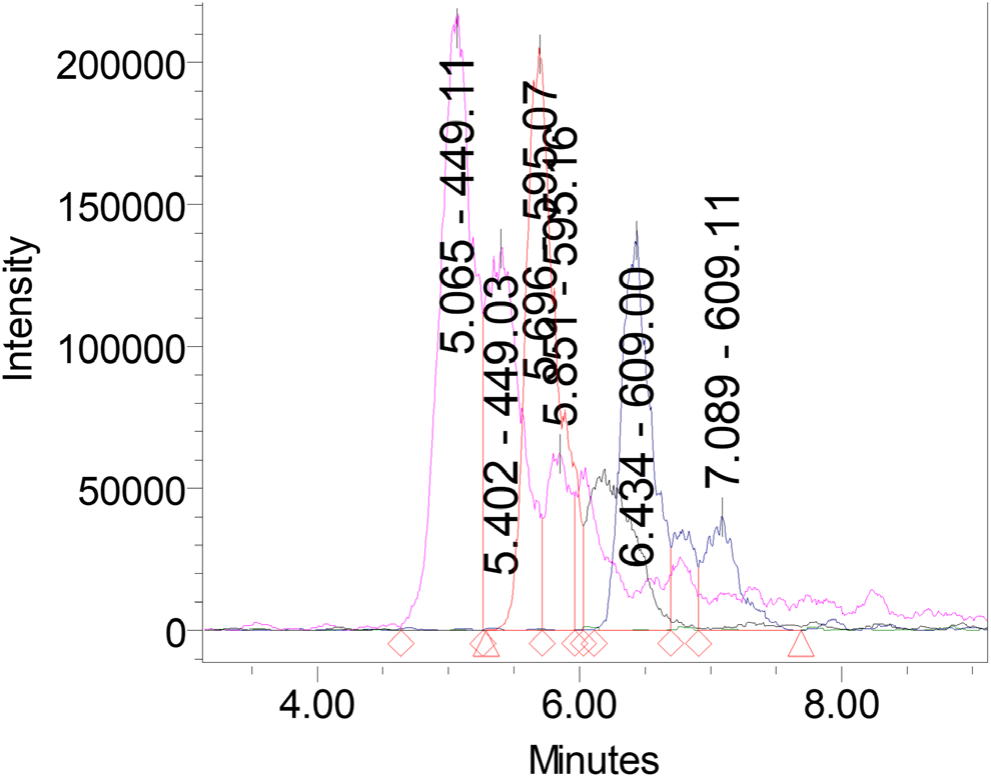
UPLC–PDA–MS chromatogram of anthocyanins in juice of *P. cerasifera* Ehrh.

Comprehensive profiling of *P. cerasifera* Ehrh. (wild *tkemali*) extracts by UPLC–PDA–MS enabled the unambiguous identification of five anthocyanin glycosides (Table 4 and Figure 3). Chromatographic separation was achieved within 6.5 min under positive electrospray ionization, yielding highly resolved peaks corresponding to cyanidin‐3‐O‐galactoside (RT 5.07 min, *m/z* 449.11 [M + H]^+^), cyanidin‐3‐O‐glucoside (RT 5.40 min, *m/z* 449.03), cyanidin‐3‐O‐rutinoside (RT 5.70 min, *m/z* 595.07), cyanidin‐3‐(6‐trans‐p‐coumaroyl)‐glucoside (RT 5.85 min, *m/z* 595.16), and peonidin‐3‐O‐rutinoside (RT 6.43 min, *m/z* 609.00). Fragmentation ions at *m/z* 286.98 and 300.91 confirmed the presence of cyanidin and peonidin aglycones, respectively. The recorded dual absorption maxima at 279.8 and 518 nm are diagnostic of flavylium chromophores, consistent with anthocyanins belonging to the cyanidin series.

**TABLE 4:** UPLC–PDA–MS characterization of anthocyanins in *P. cerasifera* Ehrh. (tkemali fruit).

Compound name	RT (min)	Molecular formula	[M + H]^+^ *(m/z)*	Main fragment *(m/z)*	UV–Vis λ_max_ (nm)
Cyanidin‐3‐O‐galactoside	5.065	C_21_H_21_O_11_	449.11	286.98	279.8; 518
Cyanidin‐3‐O‐glucoside	5.402	C_21_H_21_O_11_	449.03	286.98	279.8; 518
Cyanidin‐3‐O‐rutinoside	5.696	C_27_H_31_O_15_	595.07	286.98	279.8; 518
Cyanidin‐3‐(6‐trans‐p‐coumaroyl)‐glucoside	5.851	C_30_H_27_O_13_	595.16	286.98	279.8; 518
Peonidin‐3‐O‐rutinoside	6.434	C_28_H_33_O_15_	609.00	300.91	279.8; 518

The UPLC–PDA–MS chromatogram (Figure 3) displays the sequential elution of these pigments, with the most intense peak assigned to cyanidin‐3‐O‐rutinoside, which accounted for approximately 40%–45% of the total ion current. The remaining cyanidin‐based glycosides together contributed nearly 50%, while peonidin‐3‐O‐rutinoside comprised a smaller but distinct portion (~10%). This pattern confirms a pigment composition dominated by cyanidin derivatives, a chemotaxonomic hallmark of red‐ and dark‐red *P. cerasifera* accessions.

Comparatively, the anthocyanin profile resembles that of *P. salicina* and *P. domestica* , yet differs by the presence of cyanidin‐3‐(6‐trans‐p‐coumaroyl)‐glucoside, which is rare in cultivated plums but characteristic of stress‐adapted wild taxa. The acylated form exhibits enhanced stability due to intramolecular copigmentation, explaining the deep red–violet hue observed in *tkemali* juices and sauces (Marin‐Recinos and Pucker 2024). Moreover, the dominance of rutinosides and galactosides suggests efficient glycosylation at the 3‐hydroxyl position of the anthocyanidin nucleus—a structural adaptation that increases pigment solubility and resistance to pH fluctuations during processing.

The abundance of cyanidin glycosides underlines the high antioxidant potential of the fruit, as cyanidin derivatives possess superior radical‐scavenging capacity relative to delphinidin or pelargonidin analogues (Borrás‐Linares et al. 2015).

These findings establish a molecular basis for the strong coloration and oxidative stability of Georgian *tkemali* products, positioning *P. cerasifera* as a valuable functional source of natural colorants and bioactives in fruit‐based foods.

### 3.3 Phenolic Compound Profile (Non‐Anthocyanin Fraction)

**FIGURE 4:**
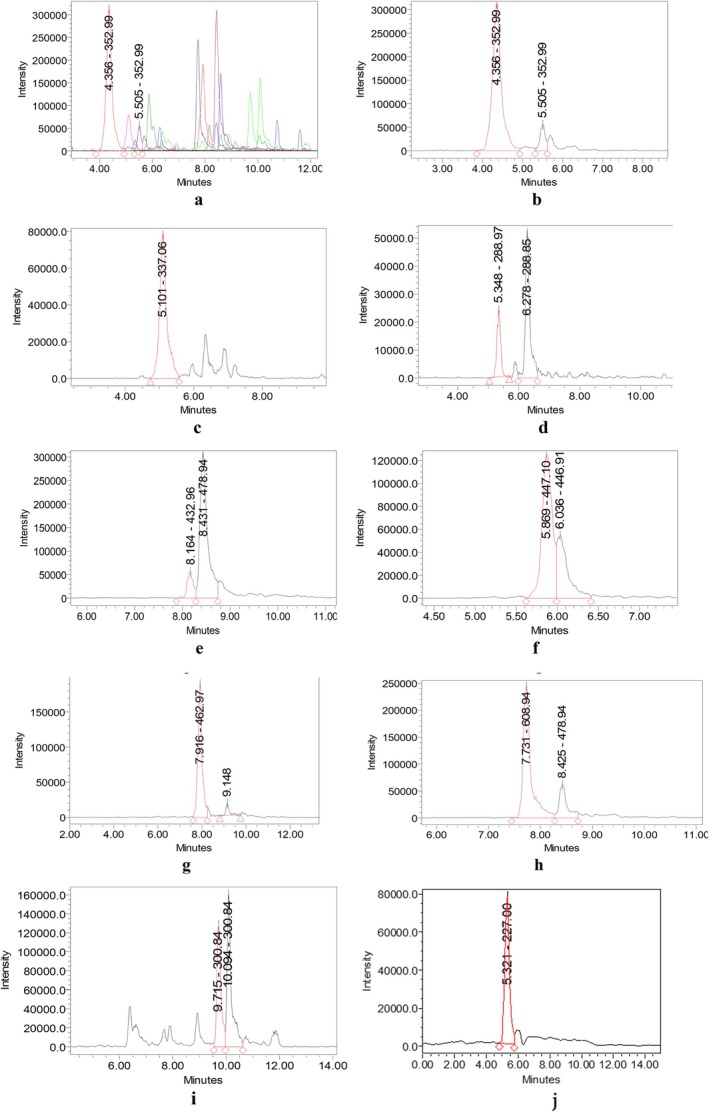
UPLC–PDA–MS chromatogram of the ethyl acetate fraction of *P. cerasifera* Ehrh. fruit extract. (a) General view of chromatograms of compounds. (b) Neochlorogenic and chlorogenic acid. (c) 3‐*p*‐Coumaroylquinic acid. (d) (+)‐Catechin and (−)‐Epicatechin. (e) Quercetin‐3‐O‐arabinoside. (f) Luteolin‐7‐O‐glucoside and Quercetin‐3‐O‐rhamnoside. (g) Quercetin‐3‐O‐glucoside. (h) Rutin (Quercetin‐3‐O‐rutinoside). (i) Quercetin (aglycone). (j) Resveratrol.

Comprehensive chromatographic profiling of the ethyl acetate fraction of *P. cerasifera* Ehrh. extracts by UPLC–PDA–MS enabled the identification of 28 non‐anthocyanin phenolic compounds spanning multiple structural classes (Table 5 and Figure 4). Detection was performed under both positive and negative ion electrospray ionization (ESI^+^/ESI^−^) modes, ensuring accurate molecular characterization. The resulting chromatogram (Figure 4) displayed a complex phenolic fingerprint between 3.4 and 11.0 min, reflecting the metabolic diversity of wild *tkemali* fruit.

**TABLE 5:** UPLC–MS (LC–ESI–MS, positive and negative ion modes) characterization of phenolic compounds in *Prunus cerasifera* Ehrh.

Compound name	RT (min)	Molecular formula	[M–H]^−^/[M + H]^+^ *(m/z)*	UV–Vis λ_max_ (nm)
Feruloylquinic acid	3.414	C_17_H_20_O_9_	366.97	324.9
*p*‐Coumaric acid	3.587	C_9_H_8_O_3_	164.16	316.8
Feruloyl‐(acetyl)‐hexose‐hexoside isomer	4.261	C_24_H_32_O_15_	558.79	310.0
Neochlorogenic acid	4.356	C_16_H_18_O_9_	353.04	324.9
Caffeoylshikimic acid	4.634	C_16_H_18_O_10_	334.98	268.7
3‐*p*‐Coumaroylquinic acid	5.101	C_16_H_18_O_8_	337.06	310.6
(+)‐Catechin	5.348	C_15_H_14_O_6_	288.97	281.7
Chlorogenic acid	5.505	C_16_H_18_O_9_	353.01	324.2
Caffeic acid hexoside	5.606	C_15_H_18_O_9_	340.98	311.2
Coumaroyl(triacetyl)‐dihexoside isomer	5.737	C_27_H_24_O_16_	613.01	268.1
Luteolin‐7‐O‐glucoside	5.869	C_21_H_20_O_11_	447.10	282.3
Coumaroyl(triacetyl)‐dihexoside isomer	5.942	C_27_H_24_O_16_	613.04	267.5
Quercetin‐3‐O‐rhamnoside	6.036	C_21_H_20_O_11_	446.91	319.9
Hexosyl‐malic acid	6.081	C_10_H_18_O_10_	296.91	282.3
(−)‐Epicatechin	6.278	C_15_H_14_O_6_	288.85	281.7
Quercetin‐3‐O‐(acetyl)‐rutinoside	6.466	C_29_H_34_O_17_	651.22	325.5
Coumaroyl(tetraacetyl)‐dihexoside isomer	6.896	C_29_H_36_O_17_	655.16	324.2
Coumaroyl(tetraacetyl)‐dihexoside isomer	7.439	C_29_H_36_O_17_	655.26	324.2
Rutin (Quercetin‐3‐O‐rutinoside)	7.731	C_27_H_30_O_16_	608.94	254.6; 351.6
Quercetin‐3‐O‐glucoside	7.916	C_21_H_20_O_12_	462.97	254.6; 352.2
Quercetin‐3‐O‐arabinoside	8.164	C_20_H_18_O_11_	432.96	254.6
Quercetin‐3‐O‐xyloside	8.431	C_20_H_18_O_11_	433.01	352.2
Hexose sugar alcohol (Sorbitol)	9.040	C_6_H_14_O_6_	180.82	212.5
Quercetin (aglycone)	9.715	C_15_H_10_O_7_	300.84	266.3
Apigenin‐7‐O‐glucoside	10.268	C_21_H_20_O_10_	430.92	266.3
Procyanidin C1	10.732	C_45_H_38_O_18_	864.81	276.3
Procyanidin B1	11.004	C_30_H_26_O_12_	576.85	278.0
Resveratrol	5.321	C_14_H_12_O_3_	227.00	282.3

The major identified constituents were grouped into four dominant phenolic subclasses:

1. **Phenolic acids**, including *neochlorogenic acid* (RT 4.36 min; *m/z* 353.04 [M–H]^−^), *chlorogenic acid* (RT 5.50 min; *m/z* 353.01), and *p‐coumaroylquinic* and *feruloylquinic acids* (RT 3.41–5.10 min; *m/z* 337–366), represented the predominant acidic fraction. These compounds are critical intermediates of the shikimate and phenylpropanoid pathways, contributing to the characteristic tart flavor and strong antioxidant properties of the fruit (Maeda and Dudareva 2012).
2. **Flavan‐3‐ols**, namely *(+)‐catechin* (RT 5.35 min; *m/z* 288.97) and *(−)‐epicatechin* (RT 6.28 min; *m/z* 288.85), were present in significant intensity, reflecting active flavanol biosynthesis typical of early‐ripening plums.
3. **Flavonols and flavones**, including *luteolin‐7‐O‐glucoside* (RT 5.87 min; *m/z* 447.10), *rutin* (RT 7.73 min; *m/z* 608.94), *quercetin‐3‐O‐glucoside* (RT 7.92 min; *m/z* 462.97), *quercetin‐3‐O‐rhamnoside* (RT 6.04 min; *m/z* 446.91), and *apigenin‐7‐O‐glucoside* (RT 10.27 min; *m/z* 430.92), were widely distributed, highlighting the diverse glycosylation patterns characteristic of *Prunus* phenolics.
4. **Stilbenes**, **exemplified** by *resveratrol* (RT 5.32 min; *m/z* 227.00), were detected in trace quantities, representing a minor yet bioactive subfraction.

The UPLC–PDA–MS chromatogram (Figure 3) exhibited clear resolution of these phenolic clusters, with UV absorption maxima between 254 and 352 nm consistent with conjugated cinnamate and flavonoid structures. The early eluting hydroxycinnamic acids (RT < 5.5 min) showed maximum absorbance near 320–325 nm, while later eluting flavonoids demonstrated dual peaks at 266 and 350 nm, typical of the *C6–C3–C6* backbone conjugation.

Quantitatively, chlorogenic and neochlorogenic acids were the most abundant hydroxycinnamates, jointly accounting for approximately 35%–40% of the total ion current, followed by catechin and epicatechin (~25%). The high proportion of caffeoylquinic derivatives indicates a robust polyphenol oxidase–regulated phenolic metabolism, possibly linked to the fruit's strong browning potential during processing (Yoruk and Marshall 2003). Moreover, the co‐occurrence of *feruloyl‐(acetyl)‐hexose‐hexoside* and *coumaroyl(tetraacetyl)‐dihexoside* isomers suggests esterification and acyl migration reactions that stabilize the phenolic matrix, enhancing color retention in processed *tkemali* products.

When compared with cultivated plums ( *P. domestica* , *P. salicina* ), wild *P. cerasifera* demonstrates a broader phenolic spectrum and higher abundance of conjugated hydroxycinnamates, reflecting both its genetic diversity and ecological adaptation to high‐altitude stress. This expanded phenolic pool contributes to the fruit's exceptional antioxidant capacity, previously corroborated by DPPH and FRAP assays in similar genotypes. The detection of *resveratrol*, albeit in low concentration, is noteworthy, as its presence in *Prunus* fruits is uncommon and indicative of induced defense metabolism under abiotic stress (Sebastià et al. 2012; Jaroszewska et al. 2025).

Collectively, these findings define *P. cerasifera* as a rich source of structurally diverse phenolic compounds, spanning the major antioxidant and copigment classes that underlie its high nutraceutical value and color stability in derivative products.

### 3.4 Mineral (Cationic) Composition of Juice

**FIGURE 5:**
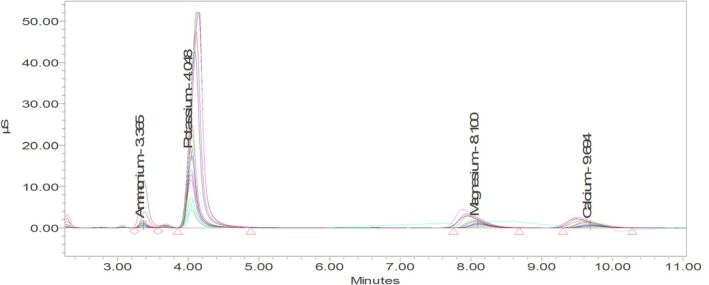
Ion‐chromatographic separation of cations (HPLC‐Conductivity detector) in *P. cerasifera* Ehrh. juice.

The mineral composition of *P. cerasifera* Ehrh. (tkemali) juice revealed a cationic profile dominated by potassium (K^+^), with substantially lower levels of magnesium (Mg^2+^) and calcium (Ca^2+^) (Table 6). Quantitative analysis using ion‐chromatography (Figure 5) showed that potassium ranged from 32.12 ± 1.28 to 155.11 ± 4.96 mg kg^−1^, accounting for more than 80% of the total measured cations. The highest K^+^ concentration was observed in the dark red Khulo 24.09 ecotype (155.11 ± 4.96 mg kg^−1^), followed by the mountain Mkvirala accession (136.53 ± 4.64 mg kg^−1^), whereas the lowest value was detected in the red Khulo 24.09 sample (32.12 ± 1.28 mg kg^−1^). Such elevated potassium levels are consistent with its physiological role in osmotic regulation, sugar translocation, and pigment biosynthesis during fruit ripening (Shen et al. 2017).

**TABLE 6:** Cation composition of *P. cerasifera* Ehrh. juice.

Sample name	Potassium (mg kg^−1^)	Magnesium (mg kg^−1^)	Calcium (mg kg^−1^)
Wild red *tkemali* (Khulo Danisparauli, 07.08)	107.70 ± 4.09	3.49 ± 0.13	8.26 ± 0.28
Wild red *tkemali* (Khulo, 24.09)	32.12 ± 1.28	0.67 ± 0.02	1.42 ± 0.05
Wild dark‐red *tkemali* (Khulo, 24.09)	155.11 ± 4.96	3.43 ± 0.12	4.30 ± 0.17
Wild dark‐red (*Khulo Gorjomi*, mountain Mkvirala)	136.53 ± 4.64	4.31 ± 0.16	7.88 ± 0.25

*Note:* Values are mean ± SD (*n* = 3).

Calcium and magnesium were present at considerably lower concentrations, 1.42–8.26 mg kg^−1^ and 0.67–4.31 mg kg^−1^ respectively. Although minor in abundance, these divalent cations are essential cofactors in pectin cross‐linking, cell‐wall stabilization, and enzymatic redox reactions. Despite their low concentrations (1.42–8.26 mg kg^−1^ Ca and 0.67–4.31 mg kg^−1^ Mg), these results are critical for maintaining fruit integrity. Calcium enhances pectin cross‐linking and cell‐wall cohesion, while both Ca and Mg regulate structural stability and reduce cracking in fleshy fruits (Hocking et al. 2016; Varaldo and Giacalone 2025).

The chromatogram in Figure 5 clearly differentiates the three major cation peaks, with retention times corresponding to potassium > calcium > magnesium. Peak intensities were proportional to their relative concentrations in Table 6, demonstrating analytical consistency across replicates. The dominance of monovalent over divalent cations suggests a high juice ionic strength and buffering capacity, potentially enhancing the stability of organic acids and anthocyanins during processing. In addition, the abundance of potassium ions contributes to the characteristic tart–salty flavor profile of tkemali.

Overall, the cationic profile of *P. cerasifera* juice highlights its nutritional and functional relevance. The high potassium content—comparable to or exceeding that of cultivated plums—confirms the species as a natural dietary source of electrolytes, while the balanced presence of Ca^2+^ and Mg^2+^ complements its overall mineral quality.

### 3.5 Bioactive Compounds and Product‐Derived Variations

#### 3.5.1 Total Phenolics, Flavonoids, Anthocyanins, and Antioxidant Activity

Marked differences were observed in the total phenolic content (TPC), flavonoids, anthocyanins, leucoanthocyanins, catechins, and DPPH radical‐scavenging capacity among *P. cerasifera* raw tissues and derived products (Table 7). The highest phenolic levels were detected in the fruit skin (14,752 ± 502 mg kg^−1^ d.m.) and in vacuum‐concentrated korao (30,962 ± 929 mg kg^−1^ d.m.), reflecting both the intrinsic enrichment of the peel and the concentration effect achieved under low‐temperature evaporation. In contrast, jam (1410 ± 51 mg kg^−1^ d.m.) exhibited the lowest TPC, largely due to thermal degradation and dilution by added sugars.

**TABLE 7:** Biologically active compounds in *P. cerasifera* Ehrh. (tkemali fruit) and derived products.

Sample	Total phenols (mg kg^−1^ d.m.)	Total flavonoids (mg kg^−1^)	Total anthocyanins (mg kg^−1^)	Leucoanthocyanins (mg kg^−1^)	Catechin (mg kg^−1^)	AA_50_ (mg sample^−1^ to inactivate 0.1 mM DPPH by 50%)
Whole fruit	10,805 ± 324	384 ± 12	656 ± 26	210 ± 8	8931 ± 268	9.56 ± 0.28
Pulp	10,679 ± 342	413 ± 14	483 ± 18	173 ± 6	7903 ± 253	8.22 ± 0.26
Skin	14,752 ± 502	267 ± 10	1663 ± 60	609 ± 21	11,889 ± 428	4.71 ± 0.16
Juice	7313 ± 278	265 ± 10	481 ± 17	163 ± 5	6216 ± 224	30.56 ± 0.97
Waste (pomace)	7766 ± 295	308 ± 11	784 ± 27	235 ± 7	6770 ± 257	9.50 ± 0.36
Purée	2109 ± 74	43 ± 2	117 ± 4	42 ± 1	1503 ± 60	11.99 ± 0.35
Jam	1410 ± 51	24 ± 1	18 ± 1	13 ± 0.5	473 ± 17	18.68 ± 0.67
Korao (classic concentrate)	16,336 ± 621	490 ± 16	2327 ± 81	435 ± 17	12,243 ± 429	2.12 ± 0.08
Korao (vacuum concentrate)	30,962 ± 929	1020 ± 26	3289 ± 105	761 ± 25	24,674 ± 716	1.12 ± 0.03
Sauce	11,392 ± 387	233 ± 8	726 ± 23	239 ± 9	8762 ± 298	4.56 ± 0.17
Tklapi (fruit leather)	5496 ± 176	182 ± 7	372 ± 11	127 ± 5	4897 ± 176	6.90 ± 0.27

*Note:* Values are mean ± SD (*n* = 3).

A similar pattern was observed for flavonoids and anthocyanins: products undergoing intense thermal treatment (jam, classic sauce) displayed 3–5‐fold reductions compared with fresh matrices, whereas vacuum concentration and minimal‐processing methods retained substantially higher pigment content. Sauce and tklapi preserved intermediate levels, indicating that shorter heating and partial dehydration mitigate losses relative to prolonged boiling.

The DPPH IC_50_ values further corroborated these trends. Vacuum korao displayed the strongest antioxidant capacity (IC_50_ = 1.12 mg per 50% inhibition), followed by classic korao (2.12 mg), skin extracts (4.71 mg), and whole fruit (9.56 mg). In contrast, juice (≈30.6 mg) and jam (≈18.7 mg) exhibited markedly weaker antioxidant performance, consistent with their reduced phenolic density. These findings confirm that low‐temperature concentration and gentle processing are key to preserving the polyphenolic integrity and antioxidant efficacy of tkemali‐derived products.

#### 3.5.2 Anthocyanin Distribution in Products

Anthocyanin profiling confirmed cyanidin‐3‐O‐galactoside, cyanidin‐3‐O‐rutinoside, and peonidin‐3‐O‐rutinoside as the principal pigments across all matrices (Table 8). The skin exhibited the highest anthocyanin concentration among raw tissues (393.7 ± 4.7 mg kg^−1^), followed by whole fruit (151.0 ± 4.5 mg kg^−1^), demonstrating the peel's dominant contribution to the pigment pool.

**TABLE 8:** Anthocyanin composition in *Prunus cerasifera* Ehrh. products.

Sample	Cy‐gal *(Cyanidin‐3‐O‐galactoside)* (mg kg^−1^)	Cy‐rut *(Cyanidin‐3‐O‐rutinoside)* (mg kg^−1^)	Peo‐rut *(Peonidin‐3‐O‐rutinoside)* (mg kg^−1^)	Total anthocyanins (mg kg^−1^)
Whole fruit	70.30 ± 2.11	57.70 ± 1.84	21.13 ± 0.84	151.01 ± 4.53
Pulp	7.55 ± 0.24	5.29 ± 0.19	2.74 ± 0.08	17.58 ± 0.59
Juice	9.27 ± 0.29	5.54 ± 0.21	2.47 ± 0.09	17.98 ± 0.53
Skin	60.31 ± 1.93	31.02 ± 1.24	135.16 ± 4.32	393.66 ± 4.73
*Tklapi* (traditional)	1.74 ± 0.05	1.97 ± 0.07	1.68 ± 0.05	5.39 ± 0.17
*Tklapi* (freeze‐dried)	9.93 ± 0.35	3.70 ± 0.10	26.87 ± 0.81	47.09 ± 1.69
*Korao* (vacuum concentrate)	7.39 ± 0.25	16.89 ± 0.64	4.81 ± 0.14	43.79 ± 1.53
*Korao* (traditional concentrate)	3.91 ± 0.15	10.24 ± 0.32	3.38 ± 0.12	41.53 ± 1.45
Jam	0.82 ± 0.02	3.36 ± 0.10	0.00	6.05 ± 0.20
*Prunus* sauce “Satsebeli A”	1.57 ± 0.06	5.06 ± 0.20	2.59 ± 0.09	15.68 ± 0.54
*Prunus* sauce “Satsebeli C”	10.47 ± 0.35	13.19 ± 0.50	2.90 ± 0.08	30.05 ± 0.96
“Satsebeli A” (freeze‐dried)	9.60 ± 0.34	14.77 ± 0.48	69.77 ± 2.13	138.82 ± 4.35

*Note:* Values are mean ± SD (*n* = 3).

Among processed products, freeze‐dried *Satsebeli A* retained the greatest anthocyanin content (138.8 ± 4.4 mg kg^−1^), followed by freeze‐dried tklapi (47.1 ± 1.7 mg kg^−1^) and vacuum korao (43.8 ± 1.5 mg kg^−1^). Traditional korao (41.5 ± 1.5 mg kg^−1^) and Satsebeli C (30.0 ± 1.0 mg kg^−1^) preserved intermediate levels, whereas heat‐intensive treatments such as boiling‐based jam (6.05 ± 0.20 mg kg^−1^) and traditional tklapi (5.39 ± 0.17 mg kg^−1^) retained less than 5% of their native anthocyanin content.

These results clearly demonstrate that anthocyanin stability is highly dependent on processing conditions: prolonged boiling and atmospheric evaporation produce extensive pigment degradation, while freeze‐drying and vacuum concentration mitigate thermal and oxidative losses. The superior retention observed in freeze‐dried Satsebeli confirms the combined protective effects of low moisture, reduced oxygen exposure, and minimal temperature stress on anthocyanin integrity.

#### 3.5.3 Phenolic Acids, Catechin, and Procyanidins

The non‐anthocyanin phenolic pool was dominated by neochlorogenic and chlorogenic acids, along with (+)‐catechin and procyanidin A_2_. Total phenolic acid + flavan‐3‐ol content ranged from 0.26 mg g^−1^ (vacuum korao) to 3.15 mg g^−1^ (freeze‐dried *Satsebeli A*), the latter representing the most phenolic‐enriched processed product (Table 9).

**TABLE 9:** Phenolic acids, catechin, and procyanidin content in *P. cerasifera* Ehrh. products.

Sample	Neochlorogenic acid (mg g^−1^)	Chlorogenic acid (mg g^−1^)	(+)‐Catechin (mg g^−1^)	Procyanidin A_2_ (mg g^−1^)	Total phenolics (mg g^−1^)
Whole fruit	0.47 ± 0.016	0.46 ± 0.017	0.20 ± 0.006	0.052 ± 0.001	1.18 ± 0.020
Pulp	0.13 ± 0.004	0.24 ± 0.009	0.03 ± 0.001	0.010 ± 0.004	0.41 ± 0.009
Juice	0.59 ± 0.023	0.79 ± 0.025	0.49 ± 0.018	0.011 ± 0.004	1.88 ± 0.029
Skin	0.21 ± 0.007	0.07 ± 0.002	0.09 ± 0.003	0.010 ± 0.004	0.38 ± 0.011
*Tklapi* (traditional)	0.09 ± 0.003	0.14 ± 0.005	0.07 ± 0.002	0.003 ± 0.0001	0.30 ± 0.009
*Tklapi* (freeze‐dried)	0.61 ± 0.023	1.35 ± 0.047	0.30 ± 0.010	0.017 ± 0.001	2.27 ± 0.033
*Korao* (vacuum concentrate)	0.09 ± 0.002	0.17 ± 0.005	0.08 ± 0.002	0.003 ± 0.0001	0.26 ± 0.009
*Korao* (traditional concentrate)	0.09 ± 0.003	0.16 ± 0.006	0.07 ± 0.002	0.003 ± 0.0001	0.25 ± 0.008
Jam	0.149 ± 0.005	0.415 ± 0.01	0.02 ± 0.001	0.003 ± 0.0001	0.57 ± 0.015
*Prunus* sauce “Satsebeli A”	0.04 ± 0.001	0.11 ± 0.003	0.02 ± 0.001	0.003 ± 0.0001	0.15 ± 0.004
*Prunus* sauce “Satsebeli C”	1.32 ± 0.050	1.02 ± 0.040	0.20 ± 0.007	0.003 ± 0.0001	2.41 ± 0.068
“Satsebeli A” (freeze‐dried)	1.37 ± 0.041	1.69 ± 0.050	0.07 ± 0.002	0.003 ± 0.0001	3.15 ± 0.085

*Note:* Values are mean ± SD (*n* = 3).

Freeze‐drying preserved phenolic acids are far more efficient than heat‐based methods. Freeze‐dried tklapi (2.27 ± 0.03 mg g^−1^) retained approximately sevenfold more hydroxycinnamates than traditional tklapi (0.30 ± 0.01 mg g^−1^). Similarly, freeze‐dried *Satsebeli A* exhibited more than a tenfold increase in caffeoylquinic derivatives relative to its thermally processed counterpart. The difference between vacuum and traditional korao was comparatively small (0.26 vs. 0.25 mg g^−1^), indicating that low‐pressure concentration provides modest benefit for phenolic acids, whereas freeze‐drying offers the greatest protection against thermally and oxidatively driven degradation.

These results align with the thermal lability of hydroxycinnamic acids and the superior retention of monomeric catechins under non‐oxidative, low‐moisture conditions.

#### 3.5.4 Quercetin Derivatives (Flavonols)

Flavonol quantification revealed rutin (quercetin‐3‐O‐rutinoside), quercetin‐3‐O‐glucoside, and quercetin‐3‐O‐pentoside as the dominant quercetin conjugates across all matrices (Table 10). In contrast to anthocyanins, flavonols were not peel‐enriched: the skin contained only 0.56 ± 0.02 mg g^−1^, whereas juice (3.03 ± 0.10 mg g^−1^) and whole fruit (2.93 ± 0.09 mg g^−1^) exhibited substantially higher levels.

**TABLE 10:** Quercetin derivatives in *P. cerasifera* Ehrh. products.

Sample	Rutin *(Quercetin‐3‐O‐rutinoside)* (mg g^−1^)	Quercetin‐3‐O‐glucoside (mg g^−1^)	Quercetin‐3‐O‐pentoside (mg g^−1^)	Total flavonols (mg g^−1^)
Whole fruit	1.27 ± 0.038	0.75 ± 0.029	0.91 ± 0.029	2.93 ± 0.087
Pulp	0.31 ± 0.009	0.56 ± 0.016	0.80 ± 0.027	1.67 ± 0.053
Juice	0.46 ± 0.015	1.29 ± 0.041	1.28 ± 0.046	3.03 ± 0.103
Skin	0.09 ± 0.003	0.31 ± 0.011	0.16 ± 0.006	0.56 ± 0.020
*Tklapi* (traditional)	0.13 ± 0.004	0.21 ± 0.007	0.27 ± 0.009	0.61 ± 0.023
*Tklapi* (freeze‐dried)	0.96 ± 0.038	2.75 ± 0.105	0.35 ± 0.013	4.06 ± 0.14
*Korao* (vacuum concentrate)	0.023 ± 0.001	0.152 ± 0.006	0.029 ± 0.001	0.204 ± 0.007
*Korao* (traditional concentrate)	0.018 ± 0.001	0.117 ± 0.003	1.47 ± 0.049	1.61 ± 0.06
Jam	0.32 ± 0.011	0.70 ± 0.025	0.47 ± 0.016	1.49 ± 0.06
*Prunus* sauce “Satsebeli A”	1.13 ± 0.041	0.78 ± 0.029	0.40 ± 0.015	2.31 ± 0.078
*Prunus* sauce “Sawebeli C”	0.04 ± 0.001	0.04 ± 0.001	0.10 ± 0.001	0.18 ± 0.005
“Satsebeli A” (freeze‐dried)	0.32 ± 0.012	0.31 ± 0.009	0.01 ± 0.001	0.64 ± 0.024

*Note:* Values are mean ± SD (*n* = 3).

Among processed products, freeze‐dried tklapi showed the highest total flavonol content (4.06 ± 0.14 mg g^−1^), followed by heat‐processed Satsebeli A (2.31 ± 0.08 mg g^−1^) and traditional korao (1.61 ± 0.06 mg g^−1^). Vacuum korao contained only trace amounts (0.204 ± 0.007 mg g^−1^), indicating that low‐pressure concentration favors anthocyanin retention far more than flavonol stability.

The predominance of rutinosides over glucosides suggests preferential rhamnosylation, which is associated with enhanced chemical resistance to hydrolysis during moderate processing (Yonekura‐Sakakibara et al. 2019). However, prolonged heating resulted in > 50% degradation of total flavonols in jam and sauces, consistent with cleavage of glycosidic bonds and subsequent oxidative polymerization.

## 4 Discussion

This study provides a comprehensive phytochemical and functional characterization of the Georgian Myrobalan plum, bridging its traditional culinary heritage with modern analytical and processing science. The identification of 34 distinct phenolic compounds reinforces its status as a rich natural source of bioactive molecules, in agreement with previous findings showing high phenolic diversity and antioxidant activity in *P. cerasifera* fruits and by‐products (Putkaradze, Vanidze and Kalandia 2025). The predominance of cyanidin‐based glycosides, particularly cyanidin‐3‐O‐rutinoside and cyanidin‐3‐O‐galactoside, mirrors profiles reported for other dark‐fleshed *Prunus* species such as *P. salicina* and *P. domestica* , where these pigments are key determinants of fruit coloration and antioxidant behavior (Valderrama‐Soto et al. 2021).

Of particular significance is the identification of cyanidin‐3‐(6‐trans‐p‐coumaroyl)‐glucoside—an acylated anthocyanin known to confer enhanced pigment stability through intramolecular copigmentation mechanisms. Such acylated forms have been reported in *P. spinosa* and *P. domestica* , where they contribute to greater resistance to pH and thermal degradation (Varga et al. 2017). The presence of this compound in wild Georgian *P. cerasifera* suggests a biochemical adaptation to high‐UV mountainous environments, similar to findings in other stress‐exposed *Prunus* ecotypes (Liu et al. 2020).

The tentative detection of resveratrol represents another novel aspect of this study. While well‐documented in grapes, its occurrence in *Prunus* species is rarely reported. Resveratrol biosynthesis is typically triggered by environmental or microbial stress, reflecting its role as a phytoalexin. Similar trace detections in other *Prunus* members under stress conditions have been linked to increased phenylpropanoid pathway activation (Sottile et al. 2023). The current finding thus underscores the biochemical plasticity of wild *P. cerasifera* , supporting its potential as a nutraceutical resource with unique antioxidant defense chemistry.

The high malic acid content (approximately 60%–65% of total organic acids) distinguishes Georgian Myrobalan plums from European cultivars dominated by citric acid. Previous analyses of Turkish and Chinese *P. cerasifera* varieties similarly reported malic acid as the primary organic acid, contributing to the fruit's pronounced tartness and natural microbial stability (Putkaradze, Vanidze and Kalandia 2025). From a food‐technology perspective, this strong organic acid buffer system enhances color stability and microbial safety during traditional tkemali sauce preparation, aligning with earlier studies linking malic acid concentration to prolonged product shelf‐life in plum‐based condiments (Levaj et al. 2012).

The comparative analysis between traditional and innovative processing methods reveals the most compelling technological implications. High‐heat methods, including open‐pan boiling and jam production, caused severe anthocyanin degradation (> 95%) and significant reductions in total phenolic content. These observations are consistent with documented losses in anthocyanin stability during high‐temperature drying and pasteurization of plums and cherries (Piga et al. 2003) and with similar degradation patterns reported in sour cherry juice processing (Toydemir et al. 2014). These losses arise primarily from hydrolysis of glycosidic bonds, oxidative cleavage, and polymerization—processes known to accelerate above 70°C, particularly in oxygen‐rich environments (Chen et al. 2018).

In contrast, non‐thermal and low‐temperature methods such as freeze‐drying and vacuum evaporation demonstrated superior retention of phenolic and anthocyanin compounds. Freeze‐dried tklapi retained nearly ninefold higher anthocyanin concentrations compared to sun‐dried samples, while vacuum concentration of korao yielded products enriched in both phenolic acids and antioxidant activity. Similar results have been observed in *P. cerasus pomace*, where freeze‐drying preserved up to seven times more anthocyanins than oven drying (Ciccoritti et al. 2018), and in *P. domestica* *prunes*, where reduced‐temperature dehydration enhanced antioxidant retention and minimized flavonol loss (Dowling 2014).

This evidence supports the conclusion that thermal degradation of phenolics follows a predictable first‐order kinetic model, with the rate constants highly sensitive to both temperature and oxygen exposure. The protective effect of low‐pressure environments, as seen in vacuum and freeze‐drying, aligns with established literature on the role of sublimation and oxygen exclusion in preserving anthocyanin structure (Drăghici‐Popa et al. 2023).

Furthermore, the high potassium content observed in this study corroborates prior findings identifying *P. cerasifera* as a potassium‐rich fruit (Jurić et al. 2025). Potassium contributes not only to osmotic regulation within the fruit but also enhances sensory characteristics by modulating acid perception. Its abundance, coupled with malic acid dominance, likely underpins the distinctive tart–salty flavor of juice and confers a nutritional advantage for electrolyte balance, as previously emphasized in dietary evaluations of plum‐derived products (Wang et al. 2012).

The phenolic and anthocyanin profiles obtained in this study are consistent with those reported in wild‐type Prunus species, where high proportions of cyanidin derivatives and neochlorogenic acids serve as chemotaxonomic markers (Guimarães et al. 2014). These compounds contribute significantly to antioxidant potential, antimicrobial properties, and pigmentation stability (Cevallos‐Casals et al. 2006). The strong correlation between total phenolic content and antioxidant activity observed here mirrors results from diverse *Prunus* matrices, confirming that phenolics are the principal contributors to redox potential (Woźniak et al. 2016).

Collectively, these findings situate Georgian *P. cerasifera* within the broader phytochemical spectrum of the *Prunus* genus but highlight distinctive features—notably, resveratrol presence, acylated anthocyanins, and malic acid dominance—that distinguish it from related cultivated plums. These chemotaxonomic and functional attributes support its use as both a cultural and nutraceutical resource, aligning with current global trends toward valorizing wild fruit biodiversity for functional food innovation (Liu et al. 2020).

Finally, from an applied perspective, the preservation of anthocyanins and phenolics under non‐thermal processing positions freeze‐dried tklapi and vacuum‐concentrated korao as superior functional food ingredients. These findings parallel advances in sour cherry and plum processing, where low‐temperature stabilization improved polyphenol bioavailability and transport efficiency across intestinal cells (Toydemir et al. 2014). Future research should therefore explore bioavailability studies and sensory stability during storage to further validate the nutritional and technological promise of Georgian *P. cerasifera* –based products.

## 5 Conclusion

This study provides a comprehensive chemical and functional characterization of Georgian *Prunus cerasifera* (tkemali) and its traditionally and innovatively processed derivatives. We established a robust phytochemical fingerprint comprising 33 distinct bioactive compounds dominated by cyanidin‐based anthocyanins, hydroxycinnamic acids, flavan‐3‐ols, and quercetin glycosides. A tentative detection of resveratrol (near the analytical detection limit) adds interest to the species' nutraceutical profile and warrants targeted confirmation. The dominance of malic acid (≈70%–75% of total acidity) and potassium further differentiates *P. cerasifera* from many cultivated plums, aligning with its characteristic sharp flavor and electrolyte value.

Processing comparisons revealed striking differences between traditional heat‐based methods and innovative low‐temperature technologies. Conventional open‐pan boiling and jam preparation led to drastic losses in anthocyanins and total phenolics, often exceeding 90%–95%, confirming the vulnerability of these compounds to thermal degradation. Conversely, freeze‐drying and vacuum concentration preserved up to ninefold higher levels of anthocyanins and sevenfold higher phenolic acids, underscoring the critical role of oxygen and temperature control in maintaining bioactive integrity. These findings contribute not only to the valorization of Georgian wild plums as functional food sources but also to the broader understanding of how minimal‐processing strategies can safeguard the health‐promoting potential of phenolic‐rich fruits.

However, this study also has several limitations that should be acknowledged. First, the phytochemical analysis was primarily based on in vitro identification and quantification techniques (UPLC–PDA–MS, LC–ESI–MS), without complementary in vivo or bioavailability assessments. Although the antioxidant potential was clearly demonstrated, the actual metabolic fate and absorption efficiency of anthocyanins, flavonoids, and phenolic acids from processed *P. cerasifera* products remain unknown. Second, while resveratrol detection is noteworthy, its concentration was tentative and near the analytical detection limit.

Processing comparisons revealed clear performance gaps between traditional heat‐based methods and low‐temperature technologies. Anthocyanins were highly heat‐labile, with > 90%–95% losses in jam and traditional tklapi, whereas freeze‐drying and vacuum concentration preserved up to ~ninefold higher anthocyanin levels. For the non‐anthocyanin phenolics, freeze‐drying conferred the strongest protection (e.g., ~sevenfold higher hydroxycinnamates in freeze‐dried vs. traditional tklapi), while vacuum concentration offered modest advantages over open pan boiling. Antioxidant assays (DPPH) paralleled compositional data, with vacuum concentrates and freeze‐dried products showing the lowest IC_50_ (highest activity). Notably, while anthocyanins are peel‐enriched, flavonols were not peel‐concentrated, underscoring matrix‐ and class‐specific stability and localization.

Further confirmation using high‐resolution LC–HRMS or targeted quantification is essential to substantiate this novel finding. Third, the sensory implications of biochemical preservation were not evaluated. Understanding how freeze‐drying and vacuum concentration affect flavor, color perception, and consumer acceptability would help translate chemical findings into real‐world product development. Finally, the study focused exclusively on fruits from specific Georgian ecotypes.

Since phenolic composition and antioxidant activity in *Prunus* species are strongly influenced by genotype and environment, future research should encompass broader geographic and genetic sampling to define chemotaxonomic variability more precisely. Future work should therefore extend in several key directions. Controlled feeding or cell‐line studies are needed to evaluate the in vivo antioxidant and anti‐inflammatory efficacy of *P. cerasifera* extracts and processed products. Exploring the interactions between phenolics and other food matrix components, such as pectins, organic acids, and minerals, could clarify their influence on compound stability and bioaccessibility. Incorporating sensory analysis and shelf‐life evaluation would also help establish the optimal balance between bioactive retention and product quality. Moreover, integrating metabolomic and transcriptomic profiling could reveal the regulatory pathways underlying anthocyanin acylation and resveratrol biosynthesis in this species, offering valuable insights for selective breeding or biotechnological enhancement.

Thus, this work establishes *Prunus cerasifera* Ehrh. as a phytochemically rich and technologically versatile fruit with high potential for use in functional foods and nutraceuticals. By combining traditional Georgian food heritage with innovative processing and analytical approaches, the study lays a scientific foundation for the sustainable development and global recognition of *tkemali*‐derived products. Addressing the identified limitations through bioavailability, molecular, and sensory studies will further consolidate the role of this wild plum as both a cultural emblem and a modern functional ingredient.

## Author Contributions

**Jeiran Putkaradze:** data curation (equal), formal analysis (equal). **Maia Vanidze:** conceptualization (equal). **Sopio Ghoghoberidze:** writing – original draft (equal). **Ruslan Davitadze:** data curation (equal). **Aleko Kalandia:** supervision (equal).

## Funding

This work was supported by the Shota Rustaveli National Science Foundation, projects AP/96/13 and PHDF‐22‐2895.

## Ethics Statement

This study does not involve any human or animal testing.

## Conflicts of Interest

The authors declare no conflicts of interest.

## Data Availability

The data supporting this study's findings are available from the corresponding author upon request.
